# Baseline correction of a correlation model for improving the prediction accuracy of infrared marker‐based dynamic tumor tracking

**DOI:** 10.1120/jacmp.v16i2.4896

**Published:** 2015-03-08

**Authors:** Mami Akimoto, Mitsuhiro Nakamura, Nobutaka Mukumoto, Masahiro Yamada, Hiroaki Tanabe, Nami Ueki, Shuji Kaneko, Yukinori Matsuo, Takashi Mizowaki, Masaki Kokubo, Masahiro Hiraoka

**Affiliations:** ^1^ Department of Radiation Oncology and Image‐applied Therapy Graduate School of Medicine, Kyoto University Kyoto; ^2^ Division of Radiation Oncology Institute of Biomedical Research and Innovation Hyogo; ^3^ Department of Radiation Oncology Kobe City Medical Center General Hospital Hyogo Japan

**Keywords:** Vero4DRT, IR Tracking, correlation model, baseline drift

## Abstract

We previously found that the baseline drift of external and internal respiratory motion reduced the prediction accuracy of infrared (IR) marker‐based dynamic tumor tracking irradiation (IR Tracking) using the Vero4DRT system. Here, we proposed a baseline correction method, applied immediately before beam delivery, to improve the prediction accuracy of IR Tracking. To perform IR Tracking, a four‐dimensional (4D) model was constructed at the beginning of treatment to correlate the internal and external respiratory signals, and the model was expressed using a quadratic function involving the IR marker position (x) and its velocity (v), namely function F(x,v). First, the first 4D model, $F_{1\text{st}}\left(x,v\right)$, was adjusted by the baseline drift of IR markers (${\text{BD}}_{\text{IR}}$) along the x‐axis, as function $F^{\prime }(x,v)$. Next, ${\text{BD}}_{\text{detect}}$, that defined as the difference between the target positions indicated by the implanted fiducial markers ($P_{\text{detect}}$) and the predicted target positions with $F^{\prime }(x,v)$ ($P_{\text{predict}}$) was determined using orthogonal kV X‐ray images at the peaks of the $P_{\text{detect}}$ of the end‐inhale and end‐exhale phases for 10 s just before irradiation. $F^{\prime }(x,v)$ was corrected with ${\text{BD}}_{\text{detect}}$ to compensate for the residual error. The final corrected 4D model was expressed as $F_{\text{cor}}\left(x,v\right)=F_{1\text{st}}\{(x-{\text{BD}}_{\text{IR}}),v\}-{\text{BD}}_{\text{detect}}$. We retrospectively applied this function to 53 paired log files of the 4D model for 12 lung cancer patients who underwent IR Tracking. The 95th percentile of the absolute differences between $P_{\text{detect}}$ and $P_{\text{predict}}$ ($\left|E_{p}\right|$) was compared between $F_{1\text{st}}\left(x,v\right)$ and $F_{\text{cor}}\left(x,v\right)$. The median 95th percentile of $\left|E_{p}\right|$ (units: mm) was 1.0, 1.7, and 3.5 for $F_{1\text{st}}\left(x,v\right)$, and 0.6, 1.1, and 2.1 for $F_{\text{cor}}\left(x,v\right)$ in the left–right, anterior–posterior, and superior–inferior directions, respectively. Over all treatment sessions, the 95th percentile of $\left|E_{p}\right|$ peaked at 3.2 mm using $F_{\text{cor}}\left(x,v\right)$ compared with 8.4 mm using $F_{1\text{st}}\left(x,v\right)$. Our proposed method improved the prediction accuracy of IR Tracking by correcting the baseline drift immediately before irradiation.

PACS number: 87.19.rs, 87.19.Wx, 87.56.‐v, 87.59.‐e, 88.10.gc

## I. INTRODUCTION

Respiratory motion is one factor causing uncertainty during beam delivery when treating tumors, particularly thoracic and abdominal tumors.^1^, ^2^ If respiratory motion is not managed appropriately, the radiation field needs to be expanded to compensate for the movements, which can lead to tissue damage as more healthy tissues around the tumor are irradiated.^3^, ^4^, ^5^ Several techniques, including forced shallow breathing, breath holding, respiratory gating, and dynamic tumor tracking (DTT), have been proposed to reduce the uncertainty caused by respiratory motion.^(2)^ Of these, there has been a recent interest in DTT, which can reposition the radiation beam dynamically in accordance with the target position. An advantage of DTT is the ability to decrease the internal margin without burdening patients with holding their breath. There are both direct and indirect DTT methods.^(6)^ Direct methods detect the internal target itself or surrogates within or near the target using imaging modalities; indirect methods observe external surrogates and then deduce the internal target position from the surrogates.

Presently, two commercially available radiotherapy devices utilize indirect DTT: the CyberKnife Robotic Radiosurgery System with an integrated Synchrony Respiratory Tracking System (Accuray, Sunnyvale, CA), and the Vero4DRT system (Mitsubishi Heavy Industries, Ltd., Hiroshima, Japan, and BrainLAB AG, Feldkirchen, Germany). The Synchrony system compensates for tumor motion by moving a robotic arm based on the internal target position estimated from the movements of light‐emitting diodes (LEDs) on the chest, using a correlation model. At the beginning of treatment, the correlation model is constructed by fitting the three‐dimensional internal tumor positions to simultaneous external LED motion. The model is checked and updated regularly during treatment by acquiring additional X‐ray images.^(7)^ By contrast, the Vero4DRT has an orthogonal kV X‐ray imaging subsystem and a gimbaled X‐ray head with a compact 6 MV C‐band linac in an O‐shaped gantry (O‐ring), which provides real‐time imaging and tumor motion compensation.^8^, ^9^, ^10^ The ExacTrac system ver. 3.1 (BrainLAB AG) is integrated with the Vero4DRT.^11^, ^12^ Presently, an infrared (IR) marker‐based DTT system (IR Tracking), which is categorized as indirect, is clinically available. The Vero4DRT system predicts the future target position from the positions of IR markers in the anterior–posterior (AP) direction on the abdominal wall using a correlation model [in this paper, four‐dimensional (4D) model].^11^, ^12^, ^13^ The 4D model is expressed as a quadratic function involving the IR marker position and its velocity with five parameters.^12^, ^13^ The Vero4DRT system cannot update the 4D model periodically during a treatment session; however, users can monitor the internal fiducials and their predicted positions during beam delivery as a benchmark for an update of the 4D model. When the predicted positions of the fiducials systematically deviate from their internal positions before beam delivery, the 4D model should be updated.^(13)^

In the indirect DTT approach, the use of external surrogates creates additional error in target prediction because it does not provide real‐time information on the internal position.^(6)^ Thus, a key issue in indirect DTT is the prediction accuracy of the correlation model for predicting the target position. Several researchers have reported that indirect DTT systems such as the Synchrony and Vero4DRT systems can construct a highly accurate correlation model;^12^, ^13^, ^14^, ^15^, ^16^, ^17^ however, breathing patterns can vary in magnitude and period during treatment sessions, and baseline drift of respiratory signals may occur,^18^, ^19^, ^20^ which degrade the prediction accuracy. A study that tested the Synchrony system concluded that the inter‐ and intrafractional baseline drift altered the correlation between the positions of the internal and external markers.^(14)^ In another study, tumor motion and the relationship between the displacements of tumors and surrogate markers changed over most 30 min treatment fractions; such changes must be taken into account for optimal motion management.^(16)^ We previously demonstrated that baseline drift of the IR marker and target positions reduces its accuracy, although the Vero4DRT system constructs a highly accurate 4D model; in addition, the baseline drift of the external IR marker positions showed weak correlation with that of the internal target positions.^(13)^ In clinical practice, a common approach to compensate for baseline drift is to update the 4D model; however, this approach increases imaging dose.^(21)^ Accordingly, a method that updates the 4D model just before beam delivery is desirable to improve the prediction accuracy with a minimal imaging dose.

Here, we propose such a correction method for IR Tracking. It changes several parameters from an initial 4D model, based only on the kV X‐ray images of the end‐inhale and end‐exhale phases.

## II. MATERIALS AND METHODS

### A. Patients and 4D modeling

We retrospectively analyzed 12 lung cancer patients who underwent IR Tracking between September 2011 and January 2013 [10 males, 2 females; median age 84 (range, 60–87) yrs]. Their lung tumors were located in the right middle (one patient), right lower (seven patients), and left lower (four patients) lobes. Four or five gold markers, 1.5 mm in diameter, were trans‐bronchially implanted around the lung tumor one to two weeks before treatment planning. The range of tumor motion, which was defined as the median peak‐to‐peak distance, was 0.2−5.6 mm, 0.9−6.6 mm, and 1.4−30.3 mm in the left–right (LR), AP, and superior–inferior (SI) directions, respectively. The median value of the respiratory cycle period was 2.8–6.7 s.

The patients were immobilized in the supine position using custom‐made body casts with both arms raised. Five IR markers were attached to the abdominal wall for monitoring the external respiratory signals. After setup correction based on bony anatomy, the ExacTrac subsystem integrated with the Vero4DRT constructed a 4D model to correlate the internal target and external surrogate positions. The IR and implanted gold markers were monitored synchronously for 20–40 s with an IR camera on the ceiling of the treatment room every 16.7 ms, and with an orthogonal kV X‐ray imaging subsystem every 80 or 160 ms, respectively. The sampling time of the kV X‐ray images changed to 160 ms automatically when the velocity of the IR marker motion had slowed. In total, approximately 400 image sets were acquired over 40 s. The optimal imaging angle of the O‐ring to monitor the implanted gold markers was determined with reference to a previous study.^(22)^ Using the acquired training data, the 4D model correlated the target positions indicated by the implanted gold markers (detected target positions; $P_{\text{detect}}$) in each direction with the position of the IR markers in the AP direction ($P_{\text{IR}}$). The 4D model [F(x,v)] was expressed as follows:

(1) $$F(x,v)={\text{ax}}^{2}+\text{bx}+c+{\text{dv}}^{2}+\text{ev}$$

where *x* is the position of the IR markers and *v* is its velocity. The predicted target position ($P_{\text{predict}}$) was calculated based on the 4D model. The parameters a, b, c, d, and e were optimized by minimizing the residual errors between $P_{\text{detect}}$ and the predicted target position for each IR marker ($P_{\text{predict},k}$; k=1–5) individually. The mean of $P_{\text{predict},k}$ for all IR markers was considered $P_{\text{predict}}$. In clinical practice, we monitored the implanted gold markers on orthogonal kV X‐ray images during beam delivery. $P_{\text{predict}}$ were displayed as a benchmark of the 4D model update. The 4D model update was needed when the implanted gold markers deviated systematically from the $P_{\text{predict}}$.^(13)^ The median frequency of 4D modeling was twice (range, two to four times) per treatment session, and the median elapsed time from the first 4D modeling procedure to the nth 4D modeling procedure (n=2−4) was 12 (range, 7–33) min.

### B. Correction of the baseline drift

We previously found that there were no significant correlations between baseline drift of $P_{\text{IR}}$ and $P_{\text{detect}}$ in each direction.^(13)^ This fact indicated that it was impossible to correct the predicted target positions from only the one‐dimensional baseline drift of $P_{\text{IR}}$ accurately. Meanwhile, there were high correlations between the predictive errors in the SI direction and the baseline drift of $P_{\text{IR}}$, and between the predictive errors and the baseline drift of $P_{\text{detect}}$ in the LR and AP directions.^(13)^ Consequently, both the baseline drift of $P_{\text{IR}}$ and $P_{\text{detect}}$ were used for 4D model correction in the current study.

**Figure 1:**
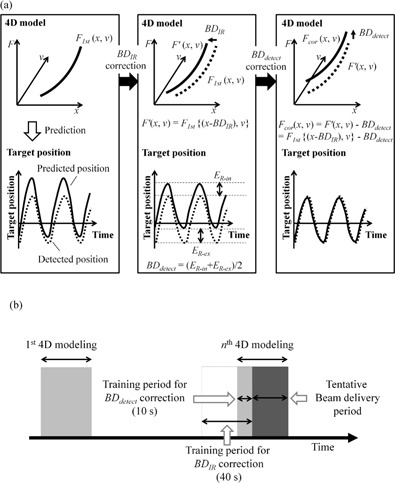
(a) Flow chart of our proposed approach for correcting the baseline drift and (b) schematic diagram of the training period for each correction. Light gray areas in Fig. 1(b) mean that the kV X‐ray beam is on during the training period, and the dark gray area means that the kV X‐ray beam is on during the tentative beam delivery period.

Figure 1(a) shows a flow chart of our proposed correction approach for correcting the baseline drift. First, the baseline of $P_{\text{IR}}$ (${\text{BD}}_{\text{IR}}$), which was defined as the median position among the peaks of $P_{\text{IR}}$ at the end‐exhale phase, was calculated during the first 4D modeling procedure and the training period for ${\text{BD}}_{\text{IR}}$ correction. The difference between these baselines was defined as the ${\text{BD}}_{\text{IR}}$ from the first 4D modeling period. Next, the first 4D model [$F_{1\text{st}}\left(x,v\right)$] was translated by the amount of ${\text{BD}}_{\text{IR}}$ along the x‐axis. Accordingly, the range of $P_{\text{predict}}$ was scaled for given range of $P_{\text{IR}}$. The corrected 4D model was expressed as $F^{\prime }(x,v)=F_{1\text{st}}${(${x‐\text{BD}}_{\text{IR}}$),v}. Subsequently, to compensate for internal residual errors resulting from baseline drift of $P_{\text{detect}}$ (${\text{BD}}_{\text{detect}}$), $P_{\text{predict}}$ was calculated from $F^{\prime }$ (x,v) at the peak positions of $P_{\text{detect}}$ of end‐inhale and end‐exhale phases in each direction separately during the training period for the ${\text{BD}}_{\text{detect}}$ correction. The peak positions of $P_{\text{detect}}$ were selectively detected around the end‐inhale and end‐exhale phases on X‐ray monitoring images. The ${\text{BD}}_{\text{detect}}$ was defined as the mean difference between $P_{\text{detect}}$ and $P_{\text{predict}}$ at end‐inhale ($E_{R‐\text{in}}$) and end‐exhale phases ($E_{R‐\text{ex}}$) (i.e., ${\text{BD}}_{\text{detect}}=\;(E_{R‐\text{in}}+E_{R‐\text{ex}})/2$). The amount of ${\text{BD}}_{\text{detect}}$ was subtracted from the intercept term of $F^{\prime }(x,v)$ to compensate for the ${\text{BD}}_{\text{detect}}$. The final corrected 4D model was expressed as $F_{\text{cor}}\left(x,v\right)=F_{1\text{st}}\{(x‐{\text{BD}}_{\text{IR}}),v\}‐{\text{BD}}_{\text{detect}}$. The Vero4DRT can monitor the internal fiducial motion every 1 s during DTT irradiation; however, the use of data during beam delivery may lead to biased results because of coarse sampling interval of 1 s. Therefore, the datasets during 4D modeling were used to assess the validity of our proposed method. In the current study, the training period for ${\text{BD}}_{\text{IR}}$ correction was set to 40 s, which included the first 10 s during the nth 4D modeling procedure to account for temporal baseline drift. Additionally, the training period for the ${\text{BD}}_{\text{detect}}$ correction was set to the first 10 s during the nth 4D modeling procedure to fully cover at least one respiratory cycle. The last 30 s during the nth 4D modeling procedure was considered the tentative beam delivery period (Fig. 1(b)).

### C. Data analysis

The Vero4DRT system generates log files containing $P_{\text{IR}},P_{\text{predict}}$, and $P_{\text{detect}}$ after 4D modeling. A total of 53 paired log files were used to assess the validity of our proposed method. $P_{\text{predict}}$ was calculated during the tentative beam delivery period from $F_{1\text{st}}\left(x,v\right)$ and $F_{\text{cor}}\left(x,v\right)$ for each paired log file. Subsequently, the differences between $P_{\text{detect}}$ and $P_{\text{predict}}$ (prediction error; $E_{p}$) were calculated from $F_{1\text{st}}\left(x,v\right)$ and $F_{\text{cor}}\left(x,v\right)$. The 95th percentile of absolute $E_{p}$ ($\left|E_{p}\right|$) over all treatment sessions for $F_{1\text{st}}\left(x,v\right)$ was compared with that for $F_{\text{cor}}\left(x,v\right)$ for each patient. In addition, the overall mean (M), systematic (Σ), and random (σ) errors were calculated using mean values and standard deviations of $E_{p}$ for $F_{1\text{st}}\left(x,v\right)$ and $F_{\text{cor}}\left(x,v\right)$ of each treatment session.

## III. RESULTS

**Figure 2:**
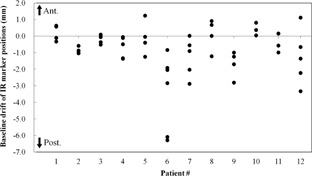
The baseline drift of IR marker positions (${\text{BD}}_{\text{IR}}$) for each patient. The baseline drifted mainly in the posterior direction (72%).

The median ${\text{BD}}_{\text{IR}}$ was −0.6 mm (range, −6.3 to 1.2 mm). Note that positive values of ${\text{BD}}_{\text{IR}}$ indicate an anterior direction. Figure 2 shows the ${\text{BD}}_{\text{IR}}$ value for each patient. Of all of the paired log files, 38 (72%) had a negative value, indicating that the baseline of the IR marker position had mainly drifted in the posterior direction. Table 1 summarizes the tumor position and the 95th percentile of $\left|E_{p}\right|$ calculated with $F_{1\text{st}}\left(x,v\right)$ and $F_{\text{cor}}\left(x,v\right)$ over all treatment sessions for each patient in the LR, AP, and SI directions. The median values were 1.0, 1.7, and 3.5 mm for $F_{1\text{st}}\left(x,v\right)$, and 0.6, 1.1, and 2.1 mm for $F_{\text{cor}}\left(x,v\right)$, respectively. It was improved by correcting the baseline drift, with the exception of two cases (Patient #4 in the AP direction, and Patient #5 in the LR direction). For all patients, the value peaked at 3.2 mm in all directions using $F_{\text{cor}}\left(x,v\right)$, compared to 8.4 mm using $F_{1\text{st}}\left(x,v\right)$.

**Figure 3:**
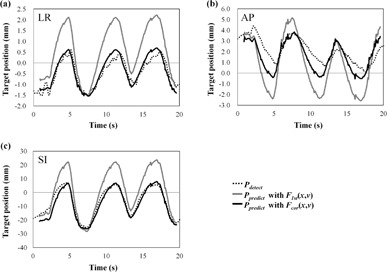
Detected target positions ($P_{\text{detect}}$) and predicted target positions ($P_{\text{predict}}$) with $F_{1\text{st}}\left(x,v\right)$ and $F_{\text{cor}}\left(x,v\right)$ in the (a) LR, (b) AP, and (c) SI directions for Patient #6 who had the greatest decline in the 95th percentile of the absolute prediction error.

**Figure 4:**
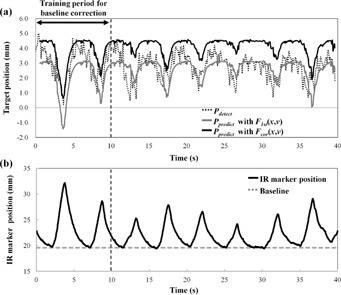
(a) Detected target positions ($P_{\text{detect}}$) and predicted target positions ($P_{\text{predict}}$) with $F_{1\text{st}}\left(x,v\right)$ and $F_{\text{cor}}\left(x,v\right)$ in the LR direction and (b) IR marker positions and their baseline during the corresponding period for Patient #5.

**Figure 5:**
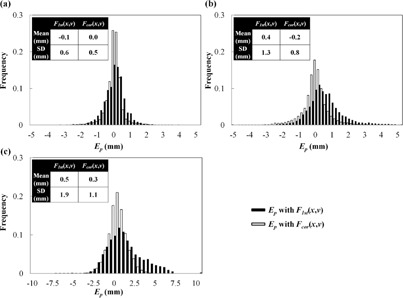
Histograms of the prediction error ($E_{p}$) with $F_{1\text{st}}\left(x,v\right)$ and $F_{\text{cor}}\left(x,v\right)$ for all patients in the (a) LR, (b) AP, and (c) SI directions.

Figure 3 shows $P_{\text{detect}}$ and $P_{\text{predict}}$ with $F_{1\text{st}}\left(x,v\right)$ and $F_{\text{cor}}\left(x,v\right)$ in Patient #6, who had the largest decline in the 95th percentile of $\left|E_{p}\right|$. The amplitude of $P_{\text{predict}}$ was similar to $P_{\text{detect}}$ with $F_{\text{cor}}\left(x,v\right)$ but larger than $P_{\text{detect}}$ with $F_{1\text{st}}\left(x,v\right)$. Figure 4(a) shows $P_{\text{detect}}$ and $P_{\text{predict}}$ with each function in the LR direction, and Figure 4(b) shows the IR marker positions and their baseline during the corresponding period for Patient #5, who had the largest increase in the 95th percentile of $\left|E_{p}\right|$. The waveform of the $P_{\text{detect}}$ was jagged due to heartbeat. In this patient, the baseline of the internal target positions drifted during the 4D modeling procedure, even though the baseline of the external IR marker positions had not drifted. Figure 5 shows histograms of $E_{p}$ with each function for all patients in the LR, AP, and SI directions. Correcting the baseline drift decreased $\left|E_{p}\right|$, with the $E_{p}$ of $F_{\text{cor}}\left(x,v\right)$ being close to zero.

The (M, Σ, σ) (units: mm) of $E_{p}$ in the LR, AP, and SI directions for $F_{1\text{st}}\left(x,v\right)$ were (0.0, 0.4, 0.3), (0.3, 0.7, 0.6), and (1.2, 1.4, 1.8), respectively. For $F_{\text{cor}}\left(x,v\right)$, they were (0.0, 0.4, 0.1), (−0.1,0.7,0.3), and (0.2, 1.0, 0.5), respectively.

**Table 1:** Tumor position and 95th percentile of absolute predictive errors for each model

		$F_{1\text{st}}\left(x,v\right)$ *(mm)*			$F_{\text{cor}}\left(x,v\right)$ *(mm)*		
*Patient #*	*Tumor Position*	*LR*	*AP*	*SI*	*LR*	*AP*	*SI*
1	RLL	0.4	1.8	2.0	0.4	0.6	1.7
2	RLL	1.5	1.7	4.3	1.2	0.6	2.2
3	RLL	0.7	1.7	3.7	0.4	0.7	2.4
4	RLL	0.5	1.1	2.1	0.6	1.2	1.8
5	RML	1.8	2.5	1.5	2.1	1.8	1.4
6	RLL	0.5	1.8	8.4	0.3	0.7	2.3
7	LLL	1.1	4.9	2.8	0.7	2.7	2.0
8	RLL	0.9	1.2	3.2	0.5	1.0	3.2
9	LLL	2.3	3.6	4.5	2.1	2.6	2.4
10	LLL	0.5	0.7	2.4	0.2	0.4	1.0
11	RLL	1.3	1.7	3.9	0.4	1.7	2.0
12	LLL	1.0	1.2	6.2	0.7	1.1	2.7

RLL=right lower lobe; RML=right middle lobe; and LLL=left lower lobe.

## IV. DISCUSSION

Although the Vero4DRT system can construct a highly accurate 4D model for phantom and clinical studies,^11^, ^12^, ^13^ the baseline drift of the IR marker and target positions reduces its prediction accuracy.^(13)^ In the present paper, we proposed a correction method for reducing prediction errors resulting from baseline drift by compensating for the baseline drift of IR markers and residual errors between $P_{\text{predict}}$ and $P_{\text{detect}}$ at the peak positions of $P_{\text{detect}}$ around the end‐inhale and end‐exhale phases. With baseline correction, the overall mean, systematic, and random errors were reduced, compared to those with $F_{1\text{st}}\left(x,v\right)$. In addition, the 95th percentile of $\left|E_{p}\right|$ over all treatment sessions peaked at 3.2 mm in all directions using $F_{\text{cor}}\left(x,v\right)$, compared with 8.4 mm using $F_{1\text{st}}\left(x,v\right)$ (Table 1). Additionally, averaged 99th percentiles of the $E_{p}$ were 1.3, 1.6, and 3.9 mm in the LR, AP, and SI directions in currently applied clinical strategies.^(23)^ In our proposed method, we didn't distinguish between amplitude variation and baseline drift. We defined the baseline as the median position among the peaks of internal and external respiratory signals at the end‐exhale phase because it is well known that the positions at the end‐exhale phase were relatively constant.^(24)^ As shown in our results, our proposed method successfully improved the prediction error in IR Tracking; therefore, the difference between amplitude variation and baseline drift would be small.

In Patient #5, the 95th percentile of $\left|E_{p}\right|$ increased from 1.8 to 2.1 mm in the LR direction. The baseline of the detected target positions drifted during the 4D modeling procedure, even though that of the IR markers did not drift, as shown in Fig. 4. Because the training period for correction of residual errors between $P_{\text{predict}}$ and $P_{\text{detect}}$ was set to the first 10 s during the nth 4D modeling procedure, $P_{\text{predict}}$ with $F_{\text{cor}}\left(x,v\right)$ fitted closely to $P_{\text{detect}}$ during the first 10 s; however, the prediction errors increased during the last 30 s. This result indicates that it may be difficult to correct the prediction errors in the presence of a changing baseline, phase, and waveform during beam delivery. We recommend correcting the baseline drift when respiratory patterns become steady.

Our method made it possible to improve the prediction accuracy without a large increase in the imaging dose and with short processing time, compared to a complete 4D remodeling. We did this by changing several parameters of the first 4D model, based on the baseline drift of the IR marker and the residual errors between $P_{\text{predict}}$ and $P_{\text{detect}}$. Our method uses only kV X‐ray images of the end‐inhale and end‐exhale phases. If the Vero4DRT system can acquire a few orthogonal kV X‐ray images selectively around the end‐inhale and end‐exhale phases by detecting changes in the velocity of IR marker motion, our method would be applicable, possibly leading to a reduction in the imaging dose. For example, given five image sets around the end‐inhale and end‐exhale phases (10 in total for one respiratory cycle) acquired for baseline correction just before each beam delivery, approximately one‐fifth of the dose will be estimated for correcting a 4D model using our method, compared with a complete 4D modeling procedure with a training period of 40 s. This is of clinical importance from the perspective of reducing radiation exposure.^(25)^

## V. CONCLUSIONS

We proposed a correction method for the baseline drift of external IR marker and internal target positions. The 95th percentile of $\left|E_{p}\right|$ over all treatment sessions peaked at 3.2 mm in all directions using $F_{\text{cor}}\left(x,v\right)$ compared with 8.4 mm using $F_{1\text{st}}\left(x,v\right)$. Therefore, our baseline correction method conducted before beam delivery improved the prediction accuracy of IR Tracking by using kV X‐ray images of the end‐inhale and end‐exhale phases with a minimal imaging dose.

## ACKNOWLEDGMENTS

This research was supported in part by the Center of Innovation Program from Japan Science and Technology Agency, JST. Takashi Mizowaki, Masaki Kokubo, and Masahiro Hiraoka have consultancy agreement with Mitsubishi Heavy Industries, Ltd., Japan.
